# Supplementary material for: A trans-translation inhibitor that targets ribosomal protein bL12 kills Mycobacterium tuberculosis
Source: J Bacteriol. 2025 Sep 3;207(10):e00236-25. doi: 10.1128/jb.00236-25 (PMC12548414; doi:10.1128/jb.00236-25)
Supplement: Supplemental figures — Fig. S1 to S8. [file jb.00236-25-s0001.pdf]

SUPPLEMENTARY FIGURES

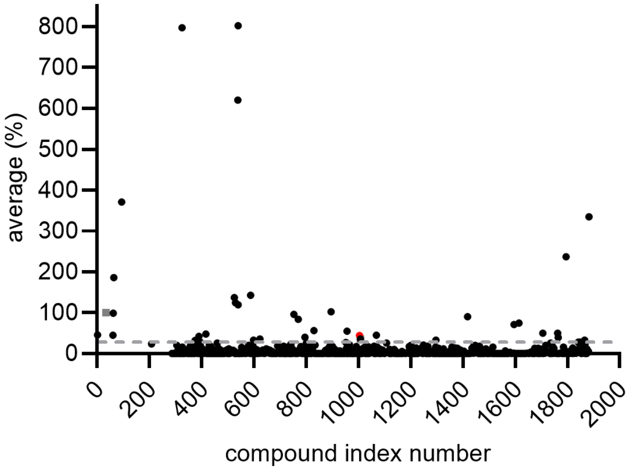

Fig. S1: Cell-based luciferase reporter screen for inhibitors of *trans*-translation. Scatter plot of the average fluorescence from the *luc-trpAt* reporter strain in the presence of 20 μM of each compound. Values from each 96-well plate were normalized to the average of 3 wells containing the negative control (DMSO) and the average of 3 wells containing KKL-35, the positive control, on the same plate. Each compound was assayed on at least two different plates, and the average normalized values are plotted. Compounds with activity one standard deviation above the median were scored as positive (dashed line). KKL-35 is shown in gray and KKL-1005 is shown in red.

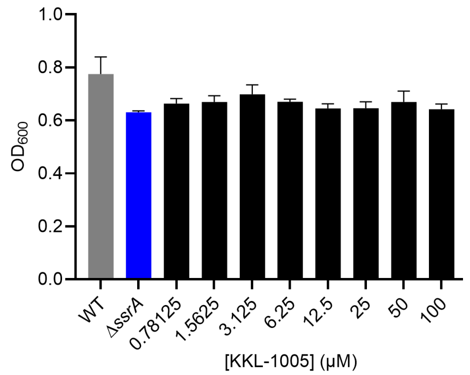

Fig. S2: KKL-1005 does not affect cell growth of *E. coli* in mCherry assays. *E. coli* Δ*ssrA* and wild-type were treated with DMSO, and the growth was compared to that of wild-type *E. coli* treated with KKL-1005. The final OD_{600} for all cultures used in Figure 1B. The mean with the error bars representing the standard deviation for three biological replicates is plotted.

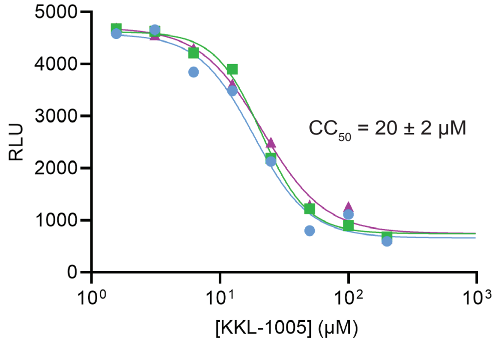

Fig. S3: KKL-1005 is cytotoxic against HeLa cells. HeLa cells were treated with KKL-1005 or DMSO. Relative luminescence units (RLU) represent the luminescence relative to that of the DMSO treated control. Data were plotted and fit to a sigmoidal function and the mean CC_{50} with standard deviation from three biological replicates is shown.

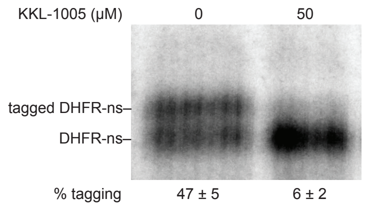

Fig. S4: KKL-1005 inhibits *E. coli trans*-translation *in vitro.* *In vitro trans*-translation assay consisting of *E. coli* components and DHFR without a stop codon was incubated with 50μM KKL-1005 or DMSO***.*** Bands corresponding to tagged and untagged DHFR are indicated, and the average percentage of DHFR protein found in the tagged band for two repeats is shown with standard deviation.

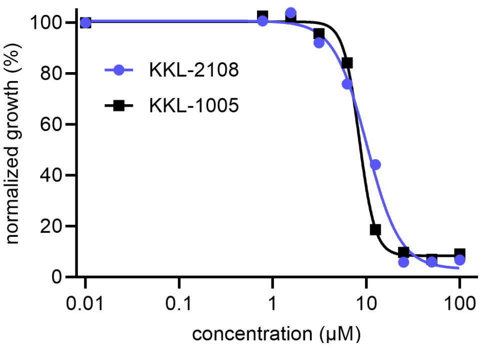

Fig. S5: Growth of *E. coli* ∆*tolC* is inhibited by both KKL-2108 and KKL-1005. The dose-response curves for the growth inhibition of *E. coli* after treatment with KKL-2108 or KKL-1005.

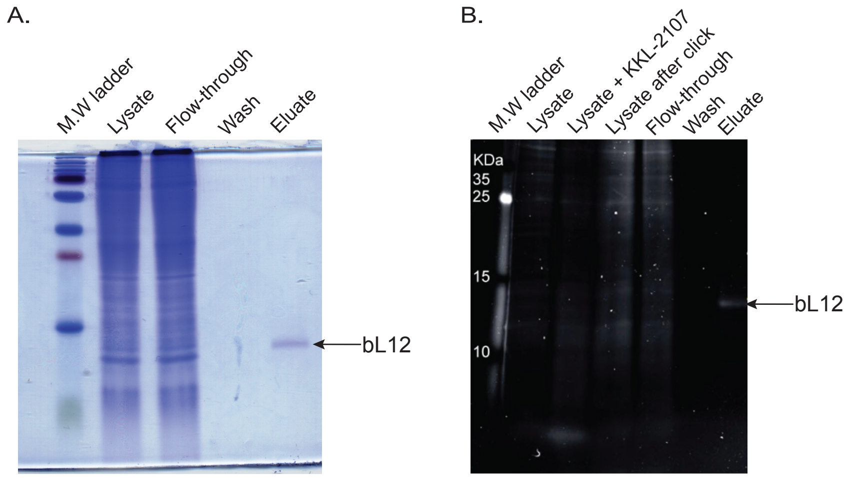

Fig. S6: KKL-1005 binds to *E. coli* bL12 *in vivo*. Click chemistry as in Fig. 3 with *E. coli* Δ*tolC.* A) Coomassie stained gel analysis of the affinity chromatography shown in Fig. 3B. C) Fluorescence scan of a gel analysis of the samples from (A) with controls for the lysate premixed with KKL-2107 only and lysate after the click reaction.

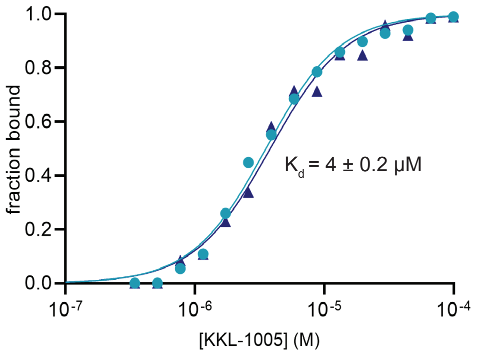

Fig. S7: KKL-1005 binds *M. tuberculosis* bL12 *in vitro*. MST was used to measure the binding of KKL-1005 with *M. tuberculosis* bL12. Change in fluorescence was measured for fluorescently labeled bL12 with KKL-1005, and the fraction of bL12 bound to KKL-1005 was calculated. Data were plotted and fit to a sigmoidal function in GraphPad Prism and the mean K_d with standard deviation from three technical replicates is shown.

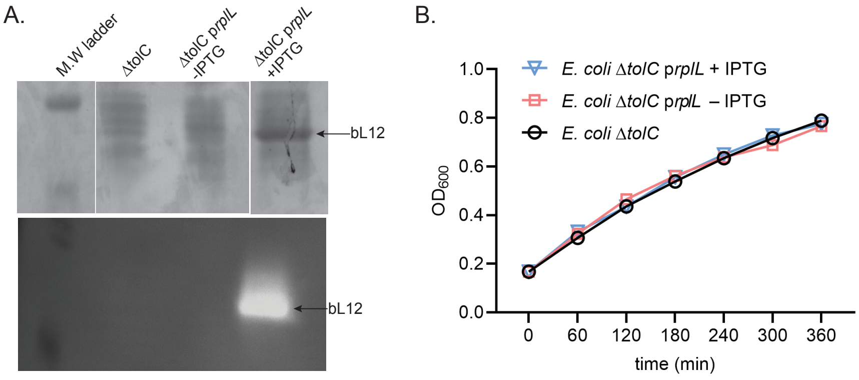

Fig. S8: Over-expression of bL12 does not interfere with the growth of *E. coli*. A) SDS-PAGE analysis of *E. coli* Δ*tolC* and *E. coli* Δ*tolC* p*rplL* lysates showing a Coomassie-stained gel (top) and a western blot (bottom) confirming over-expression of bL12 in the IPTG-induced culture. B) Growth curves for the *E. coli* Δ*tolC* and *E. coli* Δ*tolC* p*rplL* with and without IPTG.
